# Metformin and Cancer Treatment

**DOI:** 10.14740/wjon868w

**Published:** 2015-02-14

**Authors:** Annamaria Di Mari, Sebastiano Bordonaro, Sebastiano Rametta Giuliano, Fabrizio Romano, Eleonora Lanteri, Paolo Tralongo

**Affiliations:** a Medical Oncology Unit, RAO, ASP 8 Siracusa, Italy

## To the Editor

Metformin is increasingly used in the field of support therapy in cancer patients with also an impact on the natural history of cancer disease [1]. The analysis of those results offers the opportunity to bring out some considerations regarding the management of patients receiving drug combinations which include molecular targeted agents.

In the past two decades, there has been an acceleration in bringing new targeted anticancer drugs to the clinic. Most of these molecules act on those proteins that are involved in cell signaling pathways, structuring a bond that determines not only the conditioning of cell replication and apoptosis but also the functional cellular activity. In this context, it is possible to observe, in the clinical setting, a more frequent interference between biological agents and those used for the treatment of comorbidity, compared to traditional anticancer chemotherapy.

Based on cost considerations, metformin is the first drug of choice in the management of hyperglycemia in type 2 diabetes. Metformin inhibits transcription of key gluconeogenesis genes in the liver, increases glucose uptake in skeletal muscle, and decreases circulating insulin levels [2].

In cancer cells, metformin stimulates AMPK, with the inhibition of the mammalian target of rapamycin (mTOR)/ribosomal S6 kinase pathway and inhibition of pathological cell cycle progression, cell growth and angiogenesis [3, 4]. In human breast cancer cell lines, the presence of metformin determines repression of cell proliferation in both negative and positive estrogen receptor [5]. *In vitro* studies of breast cancer cell lines have demonstrated that metformin sensitizes breast cancer cells to the cytotoxic effect of chemotherapeutic drugs [6]. Diabetic patients with breast cancer receiving metformin concomitant with neoadjuvant chemotherapy have a higher pCR rate than those non-receiving metformin [7].

It has been reported that everolimus could further intensify the treatment effect [6].

Everolimus is a rapamycin analog that binds the cyclophilin FKBP-12, and this complex binds the serine-threonine kinase mTOR when it is associated with raptor and mLST8 to form a complex (mTORC1), and inhibits downstream signaling. mTORC1 lies downstream of phosphatidylinositol 3 kinase (PI3K), in a pathway that is very frequently activated in human cancers [6, 8].

Although rapamycin (with its analogues) and metformin inhibit mTOR (the former directly and the latter through AMPK signaling), there are, maybe, unrecognized differences: for example, metformin, but not rapamycin, increased exposure leading to phosphorylation of IRS-1 at Ser (789), a site previously reported to inhibit downstream signaling and to be an AMPK phosphorylated substrate under conditions of cellular energy depletion [9].

These data argue for the possibility of metformin and rapamycin analog biomolecular cross-linking whose clinical consequences are not yet fully understood.

Hence, considering the relevant therapeutic value of the everolimus alone or in combination with examestane or other agents in advanced breast cancer and in a wide spectrum of tumors, we believe that the possible metformin-everolimus impact on efficacy in diabetic cancer patients should be studied in more depth.
